# Thyroid cancer and cardiovascular diseases: a Mendelian randomization study

**DOI:** 10.3389/fcvm.2024.1344515

**Published:** 2024-04-25

**Authors:** Yamei Gao, Zhijia Wang, Jinsheng Yu, Lijun Chen

**Affiliations:** ^1^ Department of Oncology, Tianjin Binhai New Area Dagang Hospital, Tianjin, China; ^2^ Department of Cardiovascular Medicine, Tianjin Medical University General Hospital, Tianjin, China

**Keywords:** thyroid cancer, cardiovascular diseases, Mendelian randomization, the causal link, genome-wide association study

## Abstract

**Background:**

Multiple observational studies have shown associations between thyroid cancer (TC) and cardiovascular diseases (CVDs). However, the results were inconsistent, and the potential causal genetic relationship remains unclear.

**Methods:**

The genetic instruments of TC and CVDs were derived from data obtained through genome-wide association studies (GWAS). We performed the two-sample Mendelian randomization(MR) methods to investigate the causality of TC on CVDs. Summary-level statistics for CVDs, including heart failure (HF), atrial fibrillation (AF), coronary artery disease (CAD), myocardial infarction (MI), ischemic stroke (IS) and venous thromboembolism (VTE). The primary method employed in this MR analysis was the Inverse Variance Weighted (IVW) approach, and four additional algorithms were used: MR-Egger, weighted median, simple mode, and weighted mode. Additionally, we assessed the reliability of the causal relationship through pleiotropy, heterogeneity and leave-one-out sensitivity analysis.

**Results:**

In this MR analysis, we only detected causality of genetically predicted TC on HF (IVW method, odds ratio (OR) = 1.00134, 95% confidence interval (CI): 1.00023–1.00244, *p* = 0.017). However, There were no causal associations of TC with CAD, MI, AF, IS, and VTE.

**Conclusion:**

Our results confirmed the causal association between TC and HF. It is crucial to closely monitor the incidence of HF in TC patients and give comprehensive clinical intervention based on conventional treatment.

## Introduction

In recent years, epidemiological data from both domestic and international sources indicated that cancer and CVDs ranked as the top two in terms of both incidence and mortality rates (1). Many individuals diagnosed with cancer face a notable prevalence of cardiovascular events, and CVDs have emerged as a leading cause of mortality among cancer patients (2).CVDs might arise from cancer treatments such as radiation therapy, chemotherapy, targeted therapy, and other medications. Additionally, disruptions in endocrine and metabolic functions caused by cancer can contribute to these cardiovascular issues (3). If patients discontinue cancer treatment due to adverse cardiovascular events, it can further exacerbate their condition, posing a life-threatening risk. Hence, having a clear understanding of the relationship between cancer and CVDs is crucial.

Thyroid cancer (TC) is the most common malignant tumor of the endocrine system, and in recent years, its incidence has been rapidly increasing worldwide (4). In 2020 alone, there were 586,000 reported cases of TC globally, and it was anticipated to become the fourth most prevalent cancer in the United States by 2030 (5–7). Globally, CVDs are one of the leading causes of death and disability (8). It has been reported that 21.7% of TC patients’ deaths are related to coronary artery diseases (CADs) (9, 10). Many observational studies have indicated that TC patients are at increased risk of heart failure (HF), atrial fibrillation (AF), coronary artery disease (CAD), venous thromboembolism (VTE), and stroke (11–15). However, a meta-analysis revealed that the risk of HF in TC patients is lower [RR = 0.98, 95% CI (0.60–1.59)]; furthermore, after adjusting for cardiovascular risk factors, TC patients did not have a significant increase in the risk of ischemic heart disease, stroke, or HF (12). Meanwhile, Another study failed to find a significant association between a high risk of CVDs or AF and patients with TC (16). It is worth noting that these observational studies are susceptible to potential confounding factors and biases related to reverse causation. Factors such as the thyroid function status, medication side effects, and surgeries undergone by TC patients could impact the outcomes of CVDs. Therefore, It is important to note that traditional observational studies cannot elucidate the causal relationship between TC and CVDs. Therefore, establishing the causal relationship between TC and CVDs is of paramount importance.

MR serves as a robust and powerful tool for causal inference, enabling examining of the influence of exposure factors on outcomes by using genetic variants as instrumental variables (IVs) (17, 18). Due to the random assignment of alleles of IVs at conception, they remain unaffected by postnatal environmental factors, effectively mitigating concerns related to reverse causation (19). Furthermore, we can disregard study costs and ethics. Given that there was currently no evidence indicating a potential causal relationship between TC and CVDs, we carried out the two-sample MR study to investigate the causality of TC on the following six CVDs outcomes including HF, AF, CAD, MI, IS, and VTE.

## Methods

### Study design

**Figure 1:**
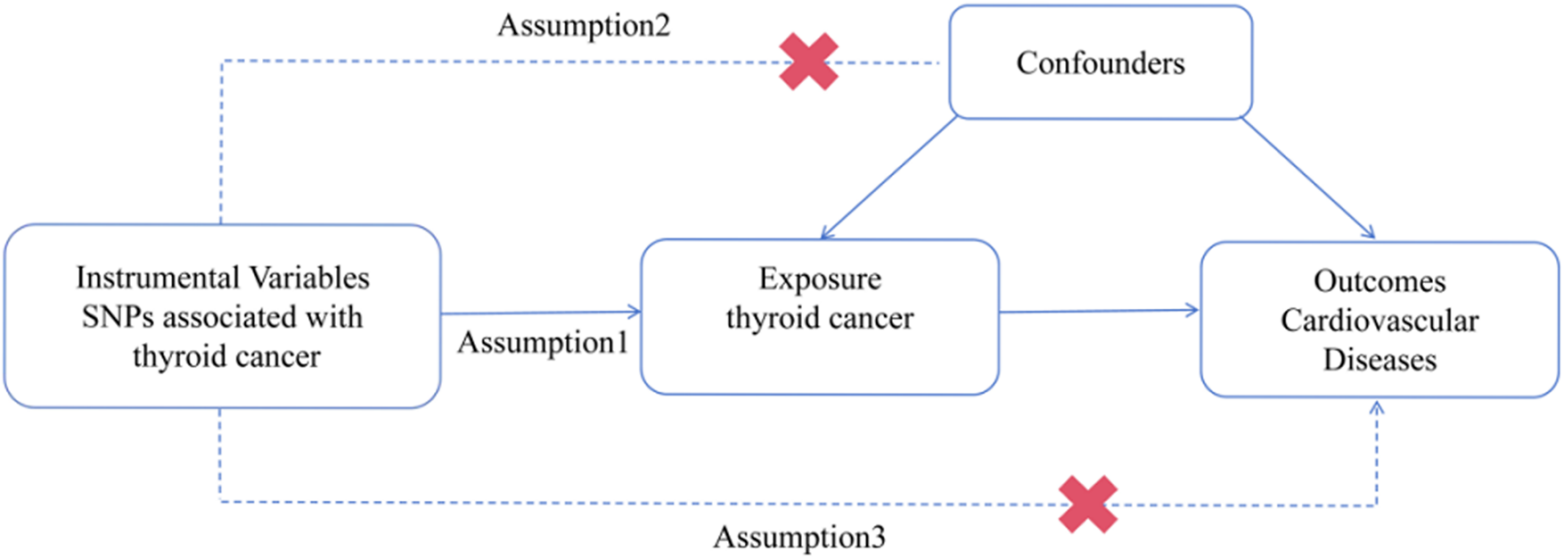
Procedures for the MR analysis of causal associations between TC and CVDs (including HF, AF, CAD, MI, IS, and VTE). SNP, single nucleotide polymorphism.

To explore the potential causal relationship between TC and CVDs, we performed MR analysis using the data obtained from GWAS summary statistics (https://www.ebi.ac.uk/gwas). The MR design is based on three assumptions: (1) the genetic variants are closely associated with TC. (2) The genetic variants are unrelated to any confounding factors. (3) The genetic variants are exclusively linked to CVDs via TC (20). The schematic diagram of the study design is illustrated in Figure 1.

### Data source

We searched for traits related to TC in a large-scale GWAS (https://gwas.mrcieu.ac.uk/datasets/ieu-a-1082), including 649 cases and 431 controls (21). For the outcome dataset, HF GWAS data were derived from the FinnGen consortium, which included 23,397 cases and 194,811 controls (https://www.finngen.fi/en). GWAS data for AF were also obtained from FinnGen consortium providing a total of 10,516 cases and 116,926 controls (https://www.finngen.fi/en).The summary dataset for CAD was derived from a public GWAS meta-analysis, providing a total of 122,733 cases and 424,528 controls (22). MI GWAS data were retrieved from the FinnGen consortium, including 12,801 cases and 205,991 controls (https://www.finngen.fi/en). Summary statistics for IS were obtained from the MEASTROKE consortium, including 7,193 cases and 406,111 controls (23). Summary level data for VTE were derived from FinnGen consortium, including 9,176 cases and 209,616 controls (https://www.finngen.fi/en). As the original studies were publicly available, it was unnecessary to apply for additional ethics consent and informed consent. The characteristics of the dataset are presented in Table 1.

**Table 1:** Details of studies included in MR analyses.

Traits	Data sources	Race	Sample size (cases/controls)	Datasets in the GWAS
Exposures				
TC	UKB	European	649/431	ieu-a-1,082
Outcomes				
HF	FinnGen	European	23,397/194,811	finn-b-I9_HEARTFAIL_ALLCAUSE
AF	FinnGen	European	10,516/116,926	finn-b-I9_AF_REIMB
CAD	CARD and UKB	European	122,733/424,528	ebi-a-GCST005195
MI	FinnGen	European	12,801/205,991	finn-b-I9_MI_EXNONE
IS	MEASTROKE	European	7,193/406,111	ebi-a-GCST006910
VTE	FinnGen	European	9,176/209,616	finn-b-I9_VTE

### Selection of IVs

We selected qualified SNPs as the genetic IVs for MR analysis. The stringent criteria for IVs are as follows: (1) Remove SNPs with linkage disequilibrium (*r*^2 ^< 0.001 within windows 10,000 kb for variants in the locus), SNPs significantly associated with TC (*p *< 5 × 10^−8^) (24); (2) Non-rare SNPs (MAF ≥ 0.01); (3)Not using SNP proxies. (4) Calculating the strength of IVs to avoid weak-tool bias: F = *R*² (N-K-1)/[K(1-*R*²)]; *R*² = {β/[se(β) * √N]}^2^ (25, 26). Here, *N* represents the sample size, and K represents the number of SNPs, we will exclude SNPs if F < 10. (5) To exclude potential pleiotropic effects, we comprehensively searched previously published literature for risk factors associated with CVDs, And by searching for SNP information on the PhenoScanner V2 website (http://www.phenoscanner.medschl.cam.ac.uk/) (27).

### Statistical analysis

All statistical analyses were conducted using the “TwoSampleMR” package in R version 4.3.1. We harmonized the aggregated SNP-TC and SNP-CVDs statistics to ensure the consistency of alleles for each SNP between TC and CVDs. And, we investigated the causal association of TC with HF, AF, CAD, MI, IS, and VTE. The primary analytical method for assessing the causal relationship is the IVW method, along with four additional algorithms, including MR-Egger, Weighted Median, Simple Mode, and Weighted Mode. *p*-value < 0.05 indicates statistical significance. MR-Egger intercept is applied to assess horizontal pleiotropy of SNPs, and if *p* < 0.05, the IVW estimate might be biased (28, 29). Cochran's Q test is performed to estimate the heterogeneity of SNPs. *P*-value indicates significant heterogeneity in the analysis results (30). Furthermore, we used the MR pleiotropy residual sum and outlier (MR-PRESSO) test to detect outliers. If outliers are identified, they will be removed, and the analysis will be re-conducted with the remaining SNPs (31). In addition, we conducted a “leave-one-out” sensitivity analysis to determine whether significant results were driven by a single SNP (32).

The associations between TC and CVDs were quantified using the OR and corresponding 95%CI. A *p*-value less than 0.05 suggests a potential causal relationship.

## Results

### Ivs

For the IVs of TC, 347 SNPs that reached the generally accepted genome-wide significance threshold (*p *< 5 × 10^−8^, *r*^2 ^< 0.001, kb = 10,000). None of these 347 SNPs were excluded as the F-statistics for each selected SNP was greater than 10 (range from 29.136 to 1,330.179). Subsequently, after assessing the SNP dataset using the PhenoScanner database, We excluded 8 SNPs associated with confounding factors: rs2566511, r1096258, rs7623609, r6546667, rs461599, rs12441088, rs8007859, and rs2157787. The remaining 339 SNPs were used for further analysis. (Detailed information can be found in Supplementary Material S1).

### The causal effect of TC on CVDs

**Figure 2:**
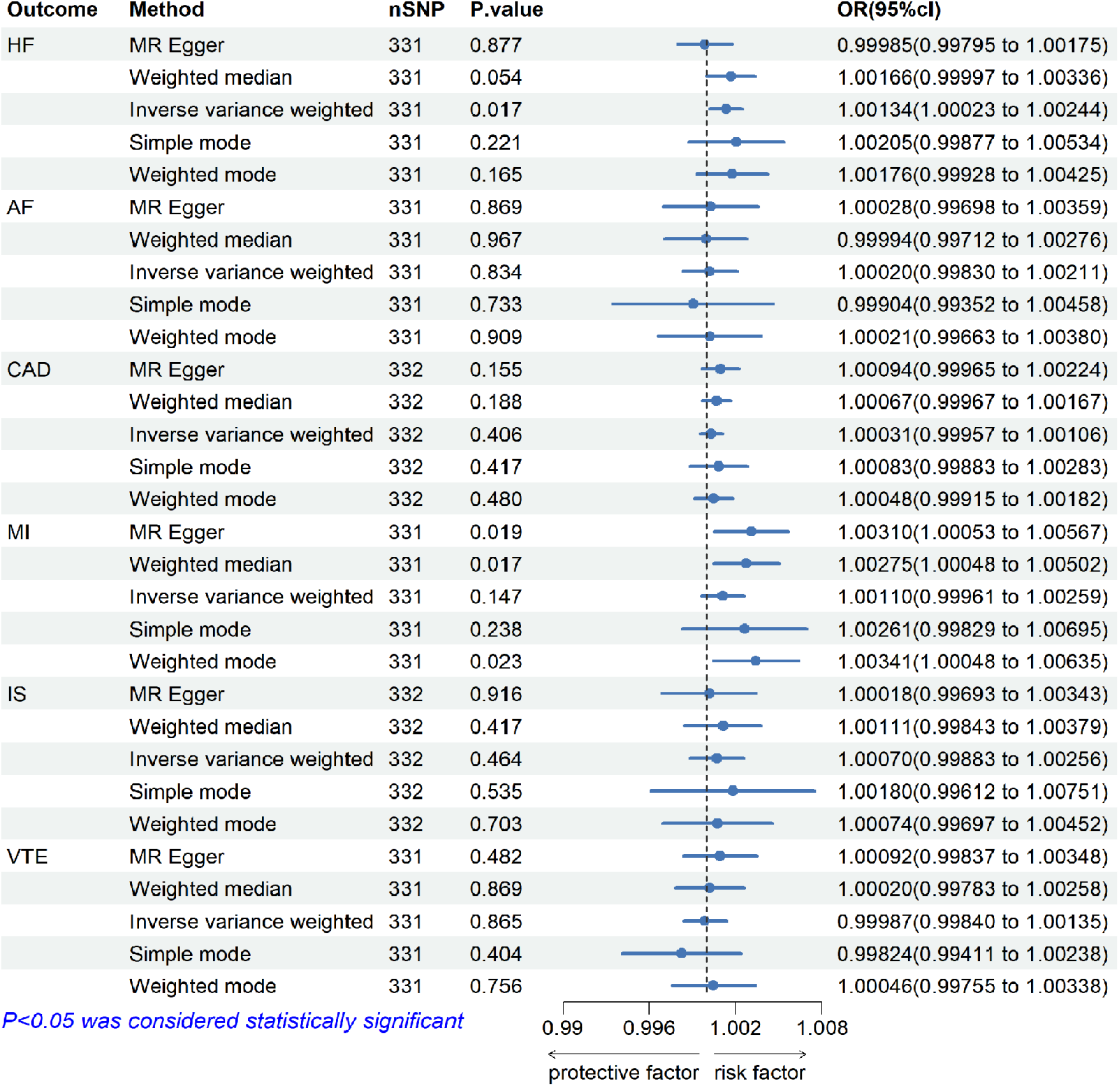
The risk association between TC and CVDs (including HF, AF, CAD, MI, IS, and VTE) in the training set visualized in a forest plot.

The results of the analysis are shown in Figure 2. The IVW method showed that higher genetically determined TC was associated with an increased risk of HF [OR = 1.00134, 95% CI (1.00023–1.00244), *p *= 0.017]. In other words, the incidence of HF in TC patients was 1.00134 times higher than in the control group. But there were no significant associations of TC with AF[OR = 1.00020, 95% CI (0.99830–1.00211), *p *= 0.834], CAD [OR = 1.00031, 95% CI (0.99957–1.00106), *p *= 0.406], MI (OR = 1.00110, 95% CI [0.99961–1.00259], *p *= 0.147), IS [OR = 1.00070, 95% CI (0.99883–1.00256), *p *= 0.464], and VTE [OR = 0.99987, 95% CI (0.99840–1.00135), *p *= 0.865].

### Sensitivity analyses of MR

The results are shown in Table 2. Sensitivity analyses include tests for pleiotropy, heterogeneity, and leave-one-out analyses. For HF, AF, CAD, MI, IS and VTE, there were no evidences of horizontal pleiotropy across the analyses in the Egger regression (*p* for intercept >0.05). In addition, We conducted IVW analysis and MR-Egger analysis to assess heterogeneity. For HF, MI, IS, and VTE, Both IVW analysis and MR-Egger analysis did not indicate the heterogeneity. However, the sensitivity analyses for TC on AF and CAD showed heterogeneity. MR-PRESSO was used to identify and remove outliers. If outliers were detected, they would be removed, and the remaining IVs would be reanalyzed. For AF, three outlier SNPs were identified: rs1889562, rs2239214, and rs8076927. After removing these three outliers, the results still indicated no causal relationship between TC and AF, with no signs of horizontal pleiotropy and heterogeneity (Detailed information can be found in Supplementary Material S2). For CAD, there is no significant outliers.

**Table 2:** Pleiotropy and heterogeneity test of the TC IVs from CVDs GWAS.

Outcomes	Pleiotropy test			Heterogeneity test					
		MR-Egger			MR-Egger			Inverse-variance weighted	
	Intercept	SE	*P*val	Q	Q_df	Q_*p*val	Q	Q_df	Q_*p*val
HF	0.002	0.001	0.062	368.023	329	0.068	371.954	330	0.055
AF	−0.000	0.002	0.957	381.579	329	0.024	381.582	330	0.026
CAD	0.000	0.001	0.248	426.128	329	0.000	427.866	330	0.000
MI	−0.003	0.002	0.062	358.96	329	0.123	362.771	330	0.104
IS	0.002	0.002	0.701	350.946	330	0.205	351.103	331	0.214
VTE	−0.002	0.002	0.327	327.263	329	0.517	328.227	330	0.517

df, degree of fredom; MR, Mendelian randomization; Q, heterogeneity statistic Q.

Scatter and funnel plots can provide a more intuitive representation of heterogeneity, as illustrated in Supplementary Figures S1, S3, S4. The leave-one-out method indicated the combined results of the remaining SNPs showed a predominantly linear trend after deleting each SNP. Thus, the robustness of our results was not influenced by any single SNP (Detailed information can be found in Supplementary Figures S2, S5–S10.

## Discussion

The MR-Egger intercept test indicated the lack of horizontal pleiotropy, suggesting that the IVs did not impact the outcome through factors unrelated to TC. The *p*-value for Cochran's Q statistic was greater than 0.05, indicating no heterogeneity among SNPs. Additionally, leave-one-out analysis suggested that the potential causal relationship between TC and the risk of HF was not driven by a single SNP. These sensitivity test results confirmed the robustness of the findings. However, we did not find sufficient evidence to support the association of TC with AF, CAD, MI, IS, or VTE.

Our results were not entirely consistent with previous studies. A observational study had shown that there was no significant increase in the risk of HF in TC patients compared to the general population [relative risk = 0.98, 95%CI(0.60–1.59)] (12). Despite this study revealed that the risk of HF in TC patients is lower. We found that in this meta-analysis, all the studied TC patients underwent thyroidectomy. Due to the intervention received by the study population, this influenced the outcome to a certain extent. This may be the reason for the inconsistency with our research results. In addition, a single-centered, cross-sectional study indicated TC patients had a significant burden of HF, which to some extent supports our research findings (11).

Two meta-analyses suggested that the risk of AF in TC was approximately 1.5 times that of the healthy control group (33, 34).This may be attributed to the Thyroidectomy as well as the iatrogenic hyperthyroid state caused by long-term suppressive therapy with thyroid hormone. Additionally, a study suggested that the risk of CAD was significantly increased in patients with TC compared to the general population (33). However, these changes were almost invariably related to alterations in thyroid function or long-term suppression of thyroid-stimulating hormone due to treatment. Besides, studies by Pajamaki et al. and Toulis et al. did not demonstrate a significant risk of CAD in patients with TC (35, 36). Qiang et al. found suggested that, following adjustments for cardiovascular risk factors, individuals with TC faced an elevated risk of AF; And however, there was no significant increase in the risk of ischemic heart disease, stroke, or HF (12). They pointed out significant heterogeneity in the study results, influenced by potential confounding factors in the original research. The impact of confounding factors such as TC treatment, thyroid hormone levels, and heterogeneity in the study population could not be ruled out.

A meta-analysis indicated a significant association between TC and a higher risk of cerebrovascular disease and AF (10). It was worth noting that the authors did not specifically indicate which cerebrovascular diseases were associated with TC. As is well known, there is an association between cancer and a higher incidence of VTE. In a case-control study, it was found that patients with TC who had distant metastasis or recent surgery had an increased risk of developing VTE (15). However, this study did not consider factors such as pregnancy, targeted therapy, systemic treatment, and radiation therapy as risk factors for VTE occurrence.

In summary,we are aware that observational studies have their limitations. Firstly, the number of eligible studies included in meta-analyses is often limited. Secondly, observational studies are susceptible to various confounding factors and biases, and their internal validity can be compromised. Thirdly, there is inevitable heterogeneity in exposure measurement, endpoint determination, statistical analysis, data collection methods, drug interventions, disease states, etc in each study. Moreover, the primary role of the thyroid is to synthesize, store, and release thyroid hormones (TH), including T3 and T4, which are regulated and controlled by thyroid-stimulating hormone (TSH). TC can lead to thyroid dysfunction, including hyperthyroidism or hypothyroidism. At the same time, there are TH receptors in myocardial and vascular tissues, and minor fluctuations in TH concentrations can affect cardiovascular physiology (37). Baumgartner et al. found that Even if thyroid hormone levels are within the normal range, an elevated FT4 level with a relatively low TSH level may still be associated with an increased risk of AF (38). However, an MR analysis did not show a causal relationship between normal-range thyroid function and CAD (39).

Compared to traditional observational epidemiological studies, MR research utilizes large-scale GWAS datasets. The MR study was conducted based on a population with European ancestry to minimize the impact of population stratification on the research results. In MR analysis, genetic variants were used as IVs, simulating, to some extent, the design of a randomized controlled trial. This approach helpes mitigate biases introduced by confounding factors and eliminates the possibility of reverse causation, thereby strengthening causal inference. Therefore, our study results have specific clinical implications. For diagnosed TC patients, clinicians should pay more attention to the potential risk of HF and intervene promptly for treatment.

However, our study has some limitations. First, to minimize the impact of population bias or heterogeneity as much as possible, the participants in the study were Europeans. However, this study did not involve trans-ethnic populations, such as East Asian populations. So the participants in the study were primarily Europeans, so the representativeness of the study results across the entire population needs further validation and the study results lack generalizability. Second, due to the complex and poorly understood biological functions of many genetic variants, we could not completely eliminate the impact of horizontal pleiotropy on the results. Third, the relatively low OR values limit their clinical relevance and guidance. Fourth, because detailed demographic information and clinical characteristics of the subjects are lacking, we are unable to perform a subgroup analysis. Finaly, regrettably, we failed to find some assumed clinical cases demonstrating the same OR level in the cardiovascular field. This will also be a focus of our future research exploration efforts.

## Conclusion

In this MR analysis-based study, we identified a potential causal relationship between TC and HF, suggesting a possible association between TC and cardiovascular health. However, our study results did not support a risk relationship between TC and other CVDs, such as AF, CAD, MI, IS, and VTE. These findings suggest that the impact of TC on cardiovascular health may be limited, but there might be a particular association with the risk of HF. Therefore, given study's limitations, we emphasize the necessity for further research to validate these results and gain a more comprehensive understanding of the relationship between TC and CVDs.

## Data Availability

The original contributions presented in the study are included in the article/**Supplementary Material**, further inquiries can be directed to the corresponding author.
