# Supplementary figures and images for: Thyroid cancer and cardiovascular diseases: a Mendelian randomization study
Source: Front Cardiovasc Med. 2024 Apr 25;11:1344515. doi: 10.3389/fcvm.2024.1344515 (PMC11080944; doi:10.3389/fcvm.2024.1344515)

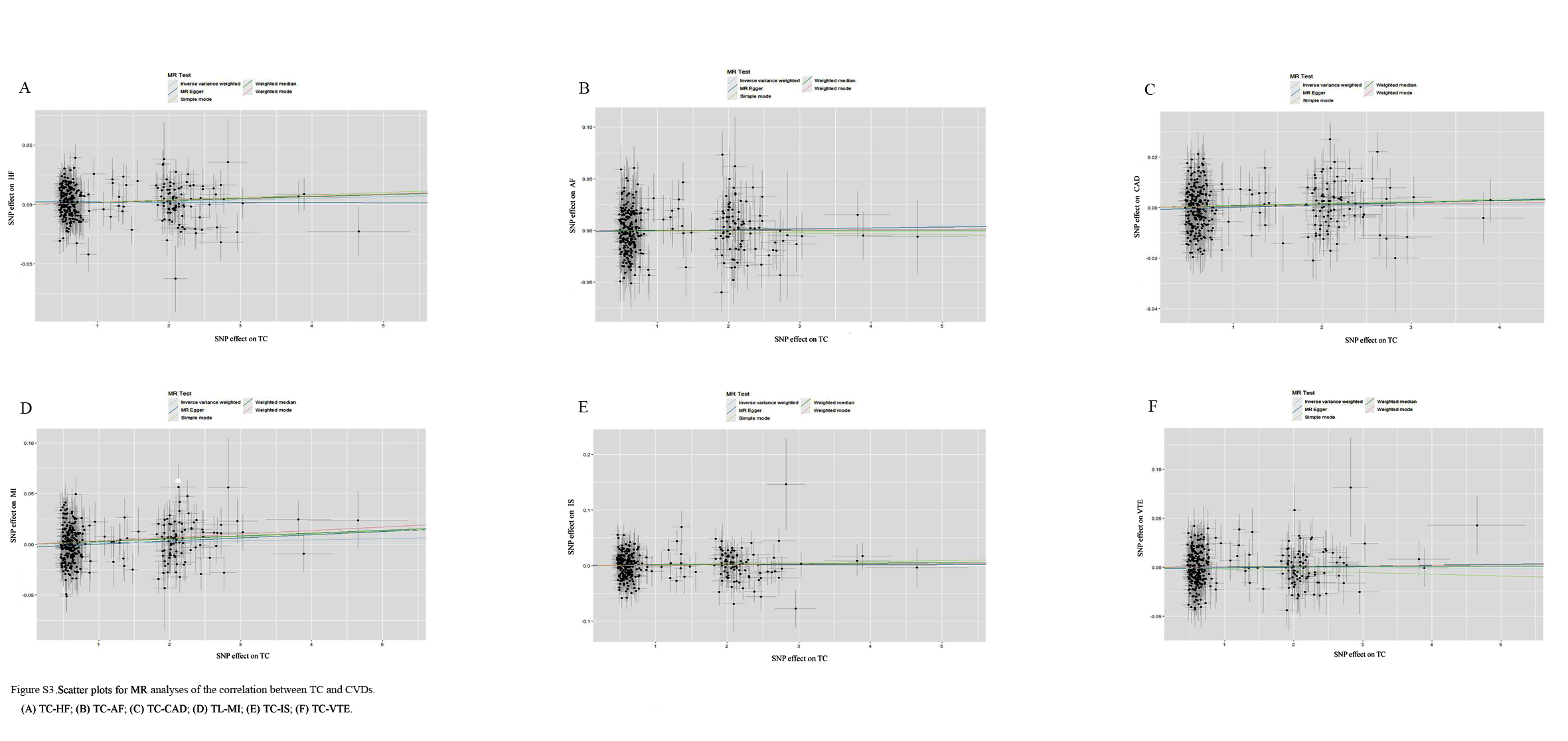

Supplement: Supplementary file 1 [file Presentation1.zip › Presentation/Supplementary Figures S1/Figure S3.tif]

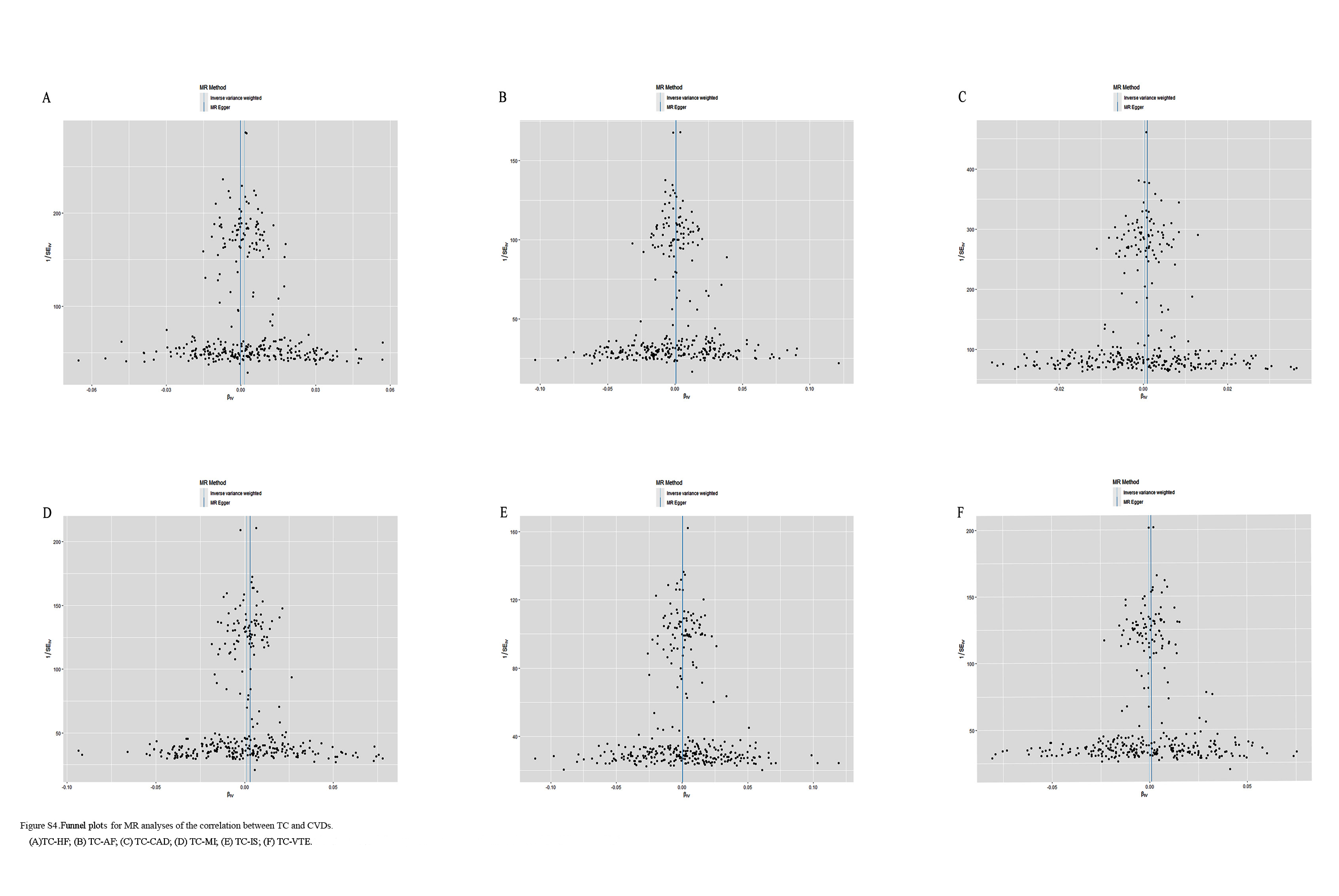

Supplement: Supplementary file 1 [file Presentation1.zip › Presentation/Supplementary Figures S1/Figure S4.tif]
